# miR-15a targets the HSP90 co-chaperone Morgana in chronic myeloid leukemia

**DOI:** 10.1038/s41598-024-65404-7

**Published:** 2024-07-02

**Authors:** Pietro Poggio, Stefania Rocca, Federica Fusella, Roberta Ferretti, Ugo Ala, Flora D’Anna, Emilia Giugliano, Cristina Panuzzo, Diletta Fontana, Valeria Palumbo, Giovanna Carrà, Daniela Taverna, Carlo Gambacorti-Passerini, Giuseppe Saglio, Carmen Fava, Rocco Piazza, Alessandro Morotti, Francesca Orso, Mara Brancaccio

**Affiliations:** 1 Department of Molecular Biotechnology and Health Sciences, University of Turin, Turin, Italy; 2 Department of Veterinary Sciences, University of Turin, Grugliasco, TO Italy; 3 Division of Internal Medicine and Hematology, San Luigi Gonzaga Hospital, Orbassano, Italy; 4 Department of Clinical and Biological Science, University of Turin, Orbassano, Italy; 5 Department of Medicine and Surgery, University of Milano-Bicocca, Monza, Italy; 6 Department of Biology and Biotechnology, Sapienza University of Rome, Rome, Italy; 7 Hematology Division and Bone Marrow Unit, Fondazione IRCCS San Gerardo dei Tintori, Monza, Italy; 8 Department of Translational Medicine (DIMET), University of Piemonte Orientale, Novara, Italy

**Keywords:** Chronic myeloid leukaemia, Chaperones, miRNAs

## Abstract

Morgana is a ubiquitous HSP90 co-chaperone protein coded by the CHORDC1 gene. Morgana heterozygous mice develop with age a myeloid malignancy resembling human atypical myeloid leukemia (aCML), now renamed MDS/MPN with neutrophilia. Patients affected by this pathology exhibit low Morgana levels in the bone marrow (BM), suggesting that Morgana downregulation plays a causative role in the human malignancy. A decrease in Morgana expression levels is also evident in the BM of a subgroup of Philadelphia-positive (Ph+) chronic myeloid leukemia (CML) patients showing resistance or an incomplete response to imatinib. Despite the relevance of these data, the mechanism through which Morgana expression is downregulated in patients’ bone marrow remains unclear. In this study, we investigated the possibility that Morgana expression is regulated by miRNAs and we demonstrated that Morgana is under the control of four miRNAs (miR-15a/b and miR-26a/b) and that miR-15a may account for Morgana downregulation in CML patients.

## Introduction

Morgana is a ubiquitous chaperone protein, essential for Drosophila and mouse development^1–3^. Morgana binds to the Heat Shock Protein 90 (HSP90)^1,2,4–8^, acting as its co-chaperone^8^, and to Rho kinase I and II (ROCKI and ROCKII), inhibiting their activity^3,9–11^. Our laboratory also demonstrated that Morgana is a component of the IKK complex, essential to sustain NF-κB activation^12,13^. Morgana expression is altered in different types of human tumors, showing overexpression or downregulation depending on the specific context^14^. Indeed, our experimental data support Morgana having both a pro-oncogenic^9,12,13,15^ and an anti-oncogenic role^3,11^. Morgana overexpression in cancer cells promotes AKT^9^ and NF-κB activation^12^ inducing cancer cell survival, invasion and metastasis formation. In addition, we recently found that the majority of cancer cells secrete Morgana in the extracellular milieu, where, in association with HSP90, it binds to surface receptors and promotes cancer cell migration^15^. On the other hand, Morgana possesses oncosuppressive functions related to its ability to bind and inhibit ROCKI and II. Indeed, in different cellular contexts, Morgana downregulation induces ROCK I and II overactivation^3,9^. ROCK I kinase activity promotes myeloid cell proliferation and survival^16,17^, while ROCK II overactivation causes centrosome overduplication and consequently multipolar mitosis, aneuploidy and karyotypic abnormalities^18–20^.

While Morgana null mice die early during embryogenesis, Morgana heterozygous mice are viable, but spontaneously develop with age a lethal and transplantable myeloproliferative disease resembling human chronic myeloid leukemia^11^. In humans, CML is primarily caused by the t(9;22)(q34;q11) translocation, leading to the formation of the Philadelphia chromosome (Ph) and the expression of the BCR-ABL fusion oncogene, responsible for driving the leukemogenic signal^21^. The onset of this leukemia is characterized by an expansion of myeloid cells at different stages of differentiation in BM and peripheral blood. In this early phase, patients affected by Ph + CML, are treated with tyrosine kinase inhibitors (TKIs) that target BCR-ABL, however, primary or secondary resistance is observed in about 20% of patients^22–24^. Genomic instability and additional chromosomal abnormalities induce Ph + CML progression from the chronic phase to blast crisis, in which TKIs are less effective^24–26^. The 5% of CML patients do not possess the BCR-ABL oncogene and are affected by atypical myeloid leukemia^27^, recently renamed MDS/MPN with neutrophilia^28,29^. The molecular basis behind the onset of this disease is complex and not completely understood. Indeed, BM cells are characterized by non-recurrent karyotypic abnormalities and mutations in several genes have been identified^30^. No standard of care is available for these patients and hematopoietic stem cell transplantation is the best option for eligible patients with appropriate donors. Otherwise, patients can be treated with hypomethylating agents, pegylated-interferon-α, hydroxyurea or erythropoiesis-stimulating agents. Despite these efforts, the median overall survival is between 12 and 25 months^10,31–33^.

Our previous research demonstrated that Morgana is expressed at very low levels in the BM cells of patients affected by MDS/MPN with neutrophilia and is robustly downregulated in 30% of Ph + CML patients in chronic phase^11^. Ph + CML and MDS/MPN with neutrophilia are both characterized by ROCK hyperactivation. In Ph + CML, ROCK is activated by BCR-ABL^16^and is further sustained by low Morgana levels^11^. In MDS/MPN with neutrophilia the decrease in Morgana expression may represent the main mechanism for ROCK overactivation^11^. Of note, treatment with fasudil, a ROCK inhibitor in clinical use, induced apoptosis in hematopoietic BM stem cell from Morgana heterozygous diseased mice and restored the efficacy of imatinib in BM cells from Ph + CML patients expressing low Morgana^11^. Analysis of exon-sequencing data of 16 patients affected by MDS/MPN with neutrophilia^11^ and search on the TCGA database on myeloproliferative neoplasms did not reveal mutations in the CHORDC1 gene. Thus, despite the clear indications supporting the role of Morgana in CML, the molecular mechanism behind the drop of its expression in patients’ BM cells is still unknown.

Here, we investigated the possibility that Morgana expression levels are controlled by microRNAs (miRNAs). miRNAs are small non-coding single-stranded RNAs^34^ that play a pivotal role in regulating gene expression, controlling a variety of cellular processes such as proliferation, survival, differentiation and metabolism and contributing to several human diseases^35^. miRNAs are involved in cancer initiation and progression both as oncogenes and tumor suppressors^36^. miRNAs can simultaneously control thousands of genes and regulate all the hallmarks of cancer, including tumor growth, apoptosis resistance, angiogenesis, invasion and metastasis formation^37–40^. We found that Morgana expression is regulated by miR-15 and miR-26 families and that miR-15a negatively correlates with Morgana expression in a cohort of MDS/MPN with neutrophilia and CML patients. Our findings suggest that, at least in a subgroup of patients, miR-15a may be responsible for the drop in Morgana expression and thus for the onset of myeloid neoplasms and resistance to therapy.

## Results

### CHORDC1 locus deletion does not account for Morgana downregulation in CML patients

As in CML cells are often present genomic abnormalities^41,42^, we hypothesized that deletion of Morgana coding gene (CHORDC1) could account for the low Morgana levels detected in the BM of patients affected by MDS/MPN with neutrophilia and in a subgroup of CML patients. We generated a fluorescent probe annealing to the CHORDC1 gene, located at 11q14.3 and we performed a FISH analysis on BM cells from patients affected by Ph + CML, with normal (four patients) and low (five patients) Morgana levels. Patient BMs were considered to express low Morgana when CHORDC1 mRNA level was less than half of the mean level observed in normal BMs used as controls (n = 10), as defined previously^11^ (Supplementary Fig. S1a). In all the cases analyzed the CHORDC1 probe showed clearly visible signals (Supplementary Fig. S1b), excluding the possibility of Morgana locus deletion. FISH analysis was also performed on BM cells from three MDS/MPN with neutrophilia cases expressing very low Morgana levels (Supplementary Fig. S1c), but also in these patients CHORDC1 gene deletion was not observed. This data indicates that Morgana locus deletion is not the major mechanism accounting for Morgana downregulation in CML patients.

### Morgana is a predicted target for miR-15a/b and miR-26a/b

**Figure 1:**
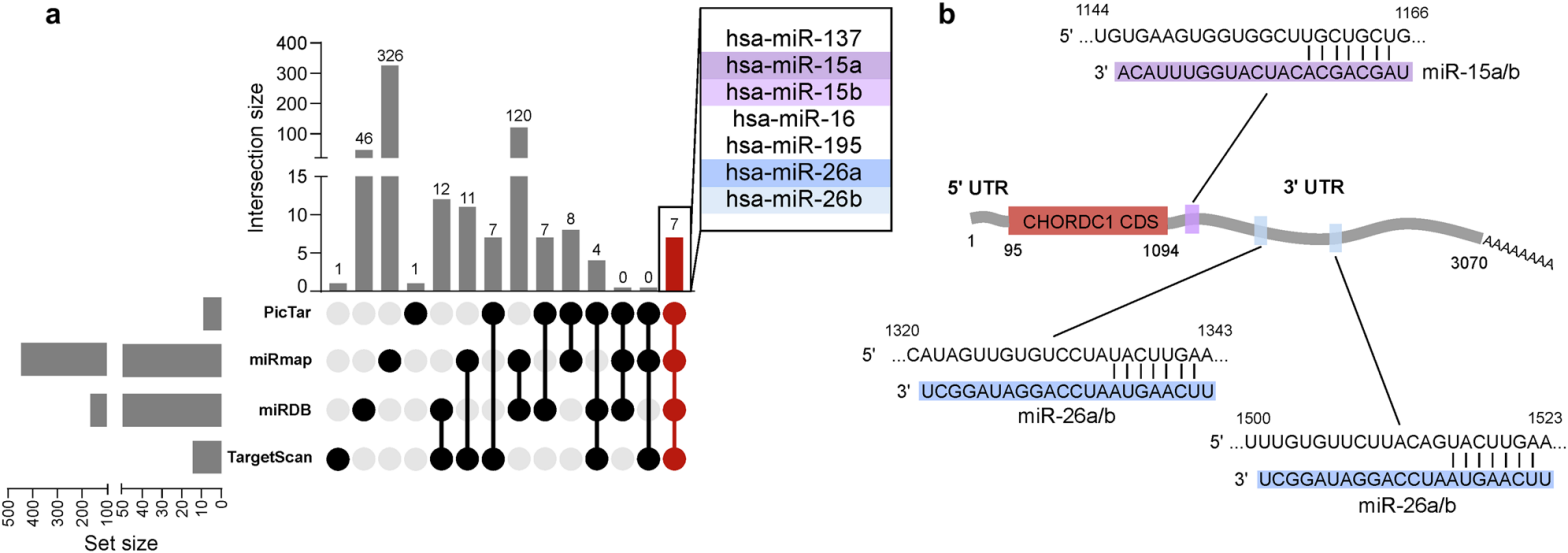
Morgana is a predicted target for miR-15a/b and miR-26a/b. (**a**) UpSet plot representing intersections between CHORDC1 targeting miRNAs predicted by 4 prediction databases (PicTar, MirMap, miRDB, TargetScan). (**b**) Scheme of Morgana transcript (Refseq: NM_012124) with the enlargement of the 3′ UTR sequences recognized by miR-15a/b and miR-26 a/b.

Several mechanisms are described to control the expression of oncosuppressors and a number of studies have shown that miRNAs and their dysregulation are implicated in the pathogenesis of hematological malignancies^43–45^. Thus, in order to identify miRNAs targeting Morgana mRNA we interrogated four miRNA target prediction databases (PicTar^46^, MirMap^47^, miRDB^48^, TargetScan^49^) (Supplementary Table S1). Among the miRNAs common to all the databases, miR-15a/b, miR-26a/b and mir-137 are the only predicted miRNAs with a conserved binding site on the CHORDC1 transcript in vertebrates. Among them, we chose to validate miR-15a/b for their well-documented role in hematological malignancies^50–52^ and miR-26a/b for having been previously described as able to target Morgana^53,54^ (Fig. 1a). Prediction databases identified one binding site for miR-15a/b and two binding sites for miR-26a/b in CHORDC1 3’UTR (Fig. 1b). We decided to validate the ability of these miRNAs to interfere with Morgana expression.

### miR-15a/b and miR-26a/b regulate Morgana mRNA and protein levels

**Figure 2:**
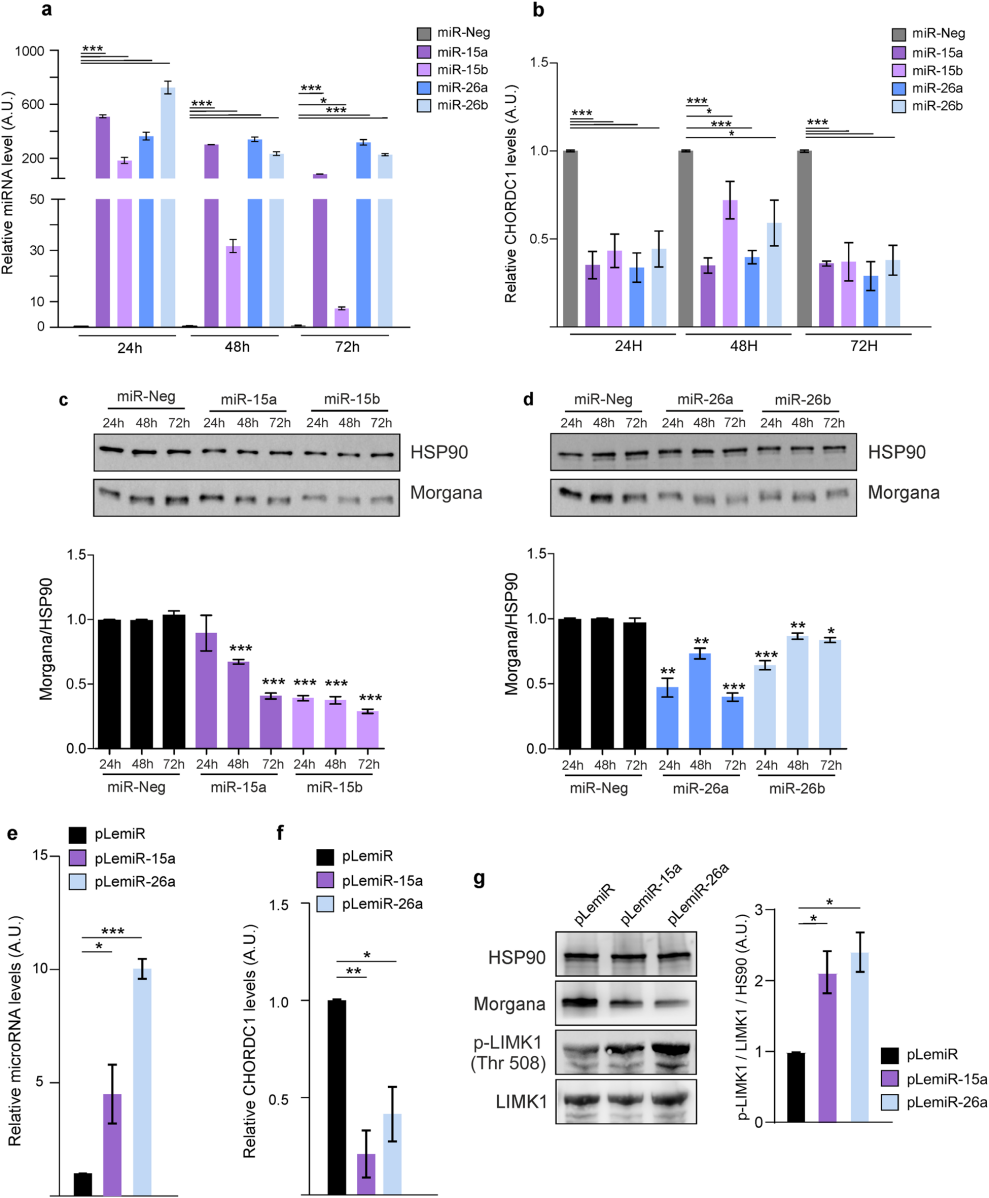
miR-15a/b and miR-26a/b regulate Morgana mRNA and protein levels. (**a**) miRNA expression levels at different time points were measured by qRT-PCR upon transfection of the indicated pre-miRs into HEK293T cells. (**b**) CHORDC1 (Morgana coding gene) mRNA expression level was assessed by qRT-PCR after transfection of the indicated pre-miRs into HEK293T cells at different time points. (**c**, **d**) Western Blot (top) and quantification graphs (bottom) assessing Morgana protein expression levels at different time points after pre-miR transfection into HEK293T cells. HSP90 was used as loading control. (**e**) Relative microRNA expression levels after infection of HEK293T cells with an empty pLemiR, or pLemiR containing miR-15a or miR-26a as assessed by qRT-PCR. (**f**) CHORDC1 mRNA expression levels measured after infection of HEK293T cells with an empty pLemiR, or pLemiR containing miR-15a or miR-26a. (**g**) Western blot analysis of HSP90, Morgana, p-LIMK1 (with relative quantification) and LIMK1 on HEK293T cells infected with an empty pLemiR, or pLemiR containing miR-15a or miR-26a. Original blots are presented in Supplementary Information. Data are the results of at least three independent experiments, bars in graphs represent mean ± S.E.M. (*P < 0.05; **P < 0.01;***P < 0.001).

We transfected HEK293T cells, which express significant amounts of endogenous Morgana, with precursors for miR-15a and miR-15b and miR-26a and miR-26b. Quantitative real-time PCR analysis showed that pre-miRNA transfection upregulated miRNA levels (Fig. 2a) and that both 15a/b and 26a/b pre-miRNAs significantly reduced CHORDC1 mRNA expression (Fig. 2b). Then, we analyzed Morgana protein levels in HEK293T cells and we found that transfections with pre-miR-15a/b and pre-miR-26a/b reduced Morgana expression by up to 50% (Fig. 2c,d). To obtain cell lines stably overexpressing miR-15 and miR-26, we infected HEK293T cells with pLemiR vectors containing pre-miR-15a or pre-miR-26a as representatives of the two miRNA families. We achieved a 5–tenfold overexpression of the miRNAs and we detected a significant downregulation of Morgana mRNA and protein (Fig. 2e–g). This modulation has a functional impact, since the phosphorylation of LIMK, a well-known ROCK substrate, increased in cells overexpressing miR-15a and miR-26a (Fig. 2g).

**Figure 3:**
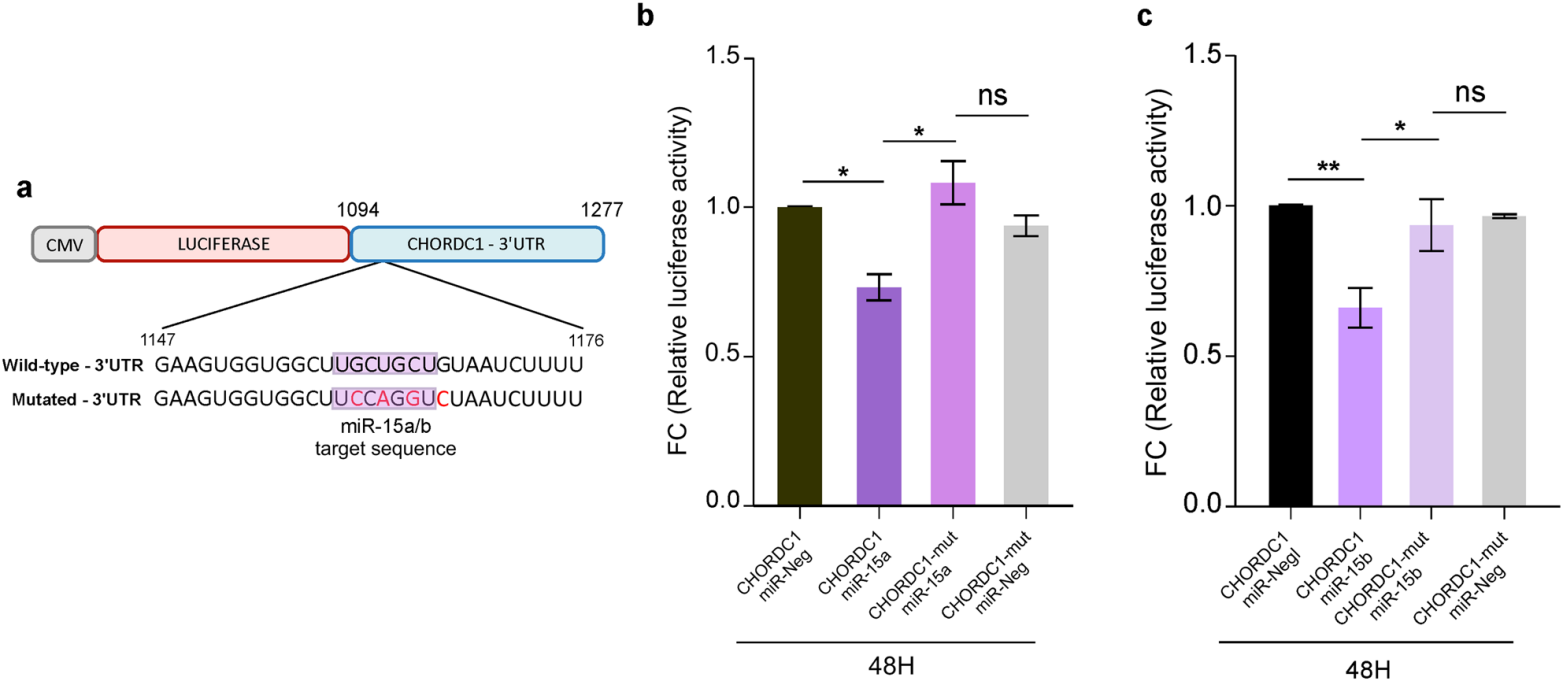
miR-15a/b directly targets CHORDC1 3’ UTR. (**a**) Scheme of the pMIR Luciferase reporter and mutations inserted in miR-15a/b target sequences of CHORDC1-3’UTR. Luciferase assays were performed 48 h post-transfection in HEK293T cells. Co-transfection was performed with pMIR reporter constructs containing either wild-type or miR-15a/b mutated target sequences, and pre-miR-15a (**b**) or pre-miR-15b (**c**) or a control precursor miRNA (miR-Neg). Data are the results of at least three independent experiments, bars in graphs represent mean ± S.E.M. (*P < 0.05; **P < 0.01).

The direct effect of miR-26 on CHORDC1 3’UTR has already been demonstrated by luciferase reporter assay and confirmed by disrupting miR-26b-mediated attenuation of luciferase activity upon mutation of the miR-26a/b seed sequences on CHORDC1 3’UTR by other groups^53^. To test the specificity of miR-15a/b in regulating Morgana expression, we cloned CHORDC1 3’UTR into a firefly luciferase reporter plasmid and mutations of the predicted seed-matching sites for both miR-15a and miR-15b were generated (Fig. 3a). Both wild-type and mutant constructs were transfected into HEK293T cells with pre-miR-15a, pre-miR-15b and a control pre-miR. Luciferase activity was significantly reduced when the wild-type CHORDC1 3’UTR was co-transfected with both pre-miR-15a and pre-miR-15b, compared to the negative control (Fig. 3b,c). This effect depends on a direct interaction between miR-15a and miR-15b and their seed sequences in CHORDC1 3’UTR, indeed their mutations completely abrogated the reduction in luciferase activity (Fig. 3b,c). Altogether, these data indicate that miR-15a/b and miR-26a/b can regulate Morgana at both mRNA and protein levels and may be responsible for Morgana downregulation in CML patients.

### miRNA 15a levels anti-correlate with Morgana expression in CML

**Figure 4:**
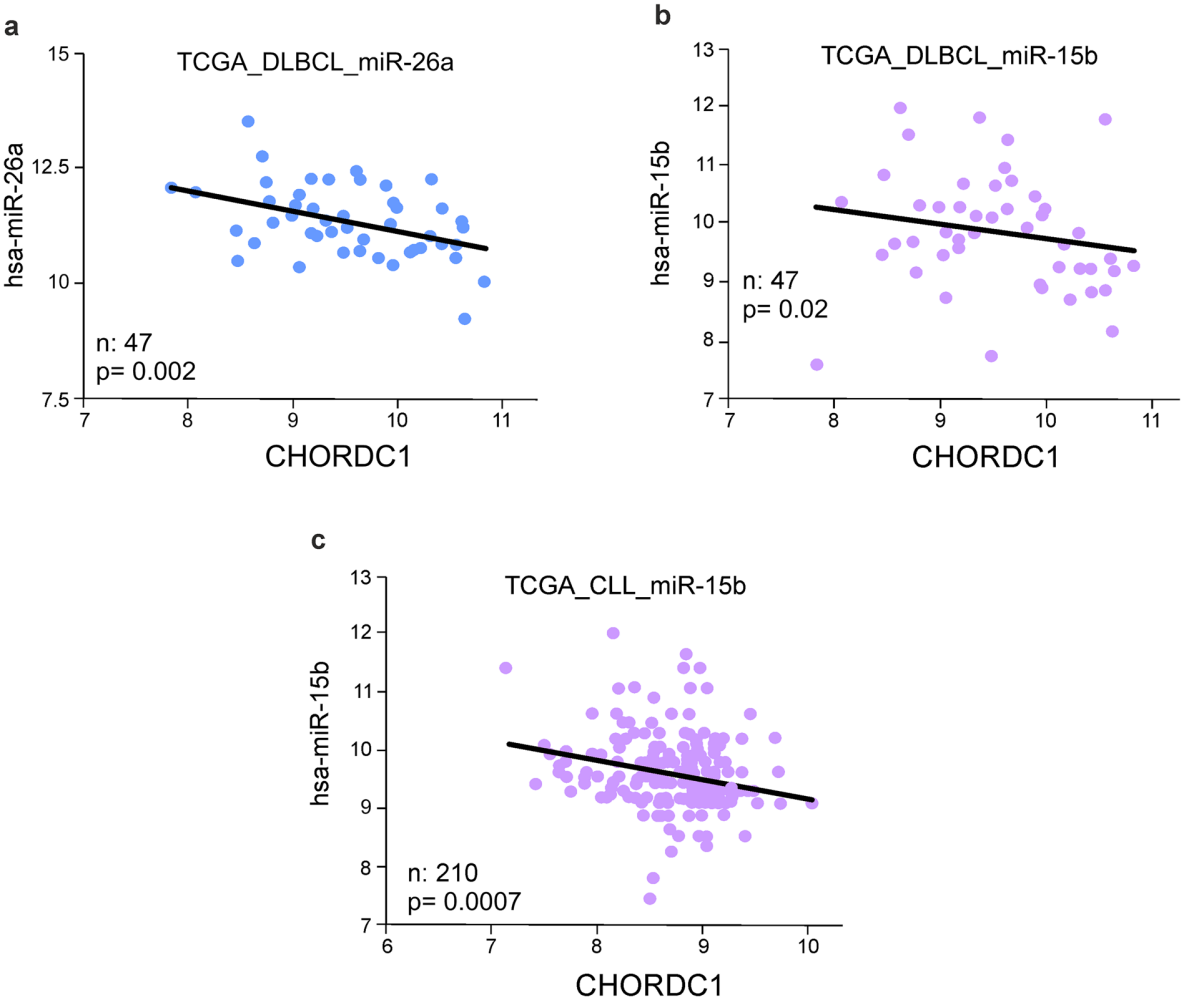
miR-26a and miR-15b negatively correlate with Morgana expression in human hematological malignancies in the TCGA database. Bioinformatic analysis indicated an anti-correlation among the indicated microRNAs and Morgana expression levels (CHORDC1 mRNA) in DLBCL (**a**-**b**) and CLL (**c**) subsets of TCGA datasets.

**Figure 5:**
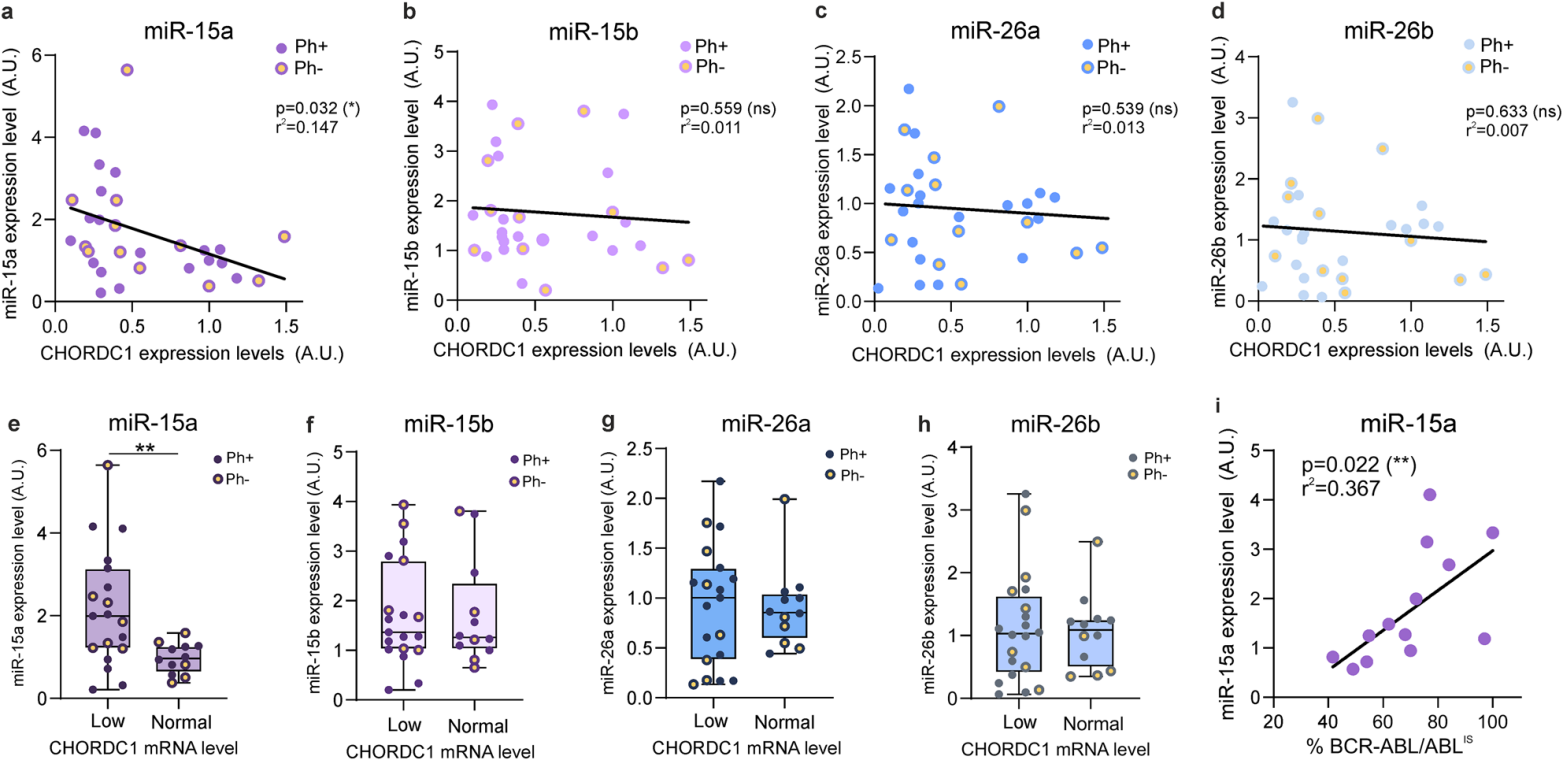
miRNA-15a levels anti-correlate with Morgana expression in patients affected by Ph + and Ph− neoplasms. (**a**, **b**, **c**, **d**) Correlation between Morgana expression (CHORDC1 mRNA assessed by qRT-PCR) and miRNA expression levels in a cohort of MDS/MPN with neutrophilia (Ph−) and Ph + CML (Ph+). (**e**, **f**, **g**, **h**) miRNA expression levels in a cohort of MDS/MPN with neutrophilia (Ph-) and Ph + CML patients (Ph+) with normal and low Morgana expression. (**i**) Correlation between BCR-ABL expression (International Scale) and miR-15a levels in Ph + CML patients. Bars in graph represent mean ± S.E.M (**P < 0.01).

To evaluate the presence of an anti-correlative relationship between Morgana and miR-15 and miR-26 families in hematological malignancies, we performed a bioinformatic analysis exploiting the Cancer Genome Atlas (TCGA) dataset. The results support our experimental findings, highlighting negative correlations between the amount of the Morgana transcript (CHORDC1) and miR-26a (Fig. 4a) and miR-15b (Fig. 4b) in 47 samples of Diffuse Large B-cell Lymphoma (DLBCL) and miR-15b in 210 Chronic Lymphocytic Leukemia (CLL) samples (Fig. 4c). These results indicate that miR-15 and miR-26 families may regulate Morgana expression levels in human hematological malignancies. To verify if the expression of miR-15 and 26 correlates with Morgana downregulation in CML patients’ BM (Supplementary Table S2), we tested, by quantitative real-time PCR, both miRNAs and CHORDC1 transcript expression in a cohort of 31 CML patients in chronic phase (12 Ph- and 19 Ph+). We found that miR-15a significantly anti-correlates with Morgana expression (Fig. 5a), while miR-15b and miR-26a/b did not show significant correlation (Fig. 5b–d). Indeed, miR-15a was significantly overexpressed in patients affected by CML and MDS/MPN with neutrophilia with low Morgana expression levels (Fig. 5e), while miR-15b and miR-26a/b levels were not different between Morgana normal and Morgana low groups (Fig. 5f–h). Of note, the expression levels of BCR-ABL and miR-15a significantly correlated, opening the intriguing possibility that BCR-ABL could potentiate miR-15a expression (Fig. 5i, Supplementary Fig. S2a–c). These results suggest that in CML, Morgana levels may depend on miR-15a expression.

## Discussion

Morgana haploinsufficiency causes the onset of a lethal myeloproliferative neoplasm in the mouse, resembling human CML and MDS/MPN with neutrophilia. This evidence indicates a causative role of Morgana downregulation in the onset of the mouse pathology. The fact that BMs from MDS/MPN with neutrophilia patients express very low Morgana levels is suggestive of equal relevance in human pathology. Morgana expression levels are also relevant in Ph + CML, where its underexpression confers imatinib resistance by inducing ROCK hyperactivation. Indeed, the combined treatment with imatinib and a ROCK inhibitor restored an apoptotic response in BM cells from CML patients with low Morgana^11^. However, the causes of Morgana downregulation in patients’ BM cells remain elusive. In this study, we addressed this issue and found that the CHORDC1 locus is not deleted in the BM from 5 CML and 3 MDS/MPN with neutrophilia patients expressing very low Morgana levels. This suggests that deletion is not the predominant mechanism through which leukemia cells suppress Morgana expression. Given that miRNA upregulation has been often described in cancer cells to promote oncosuppressor silencing^39^, we decided to investigate the relevance of miRNAs in regulating Morgana expression. We selected miR-15 and miR-26 families by interrogating four miRNA target prediction databases and demonstrated their ability to interfere with Morgana expression. Our findings are supported by unbiased studies, in which the Morgana transcript has been found among the targets of miR-26a and b^53,54^ and miR-15 and miR-16^55^. Bioinformatic analysis between Morgana expression and miR-15 and miR-26 families on the TCGA datasets revealed a significant inverse correlation with miRNAs 26a, 16a, and 15b in Diffuse Large B-cell Lymphoma and Chronic Lymphocytic Leukemia. These results suggest that these miRNAs have the potential to regulate Morgana expression in human hematological neoplasms.

miRNA-26 family has aroused interest for its role in cancer where it regulates genes involved in proliferation, survival, metabolism and invasion^56^. miRNA-26a has been found to be downregulated in melanoma^57^, breast^58^, bladder^50^ and prostate cancer^53^, but strongly overexpressed in glioma^59^ and ovarian cancer^60^. miR-26 inhibits cell viability and invasiveness in melanoma^61^, cell aggressiveness in bladder cancer^62^ and cell proliferation in ovarian cancer^63^ and promotes cell sensitivity to doxorubicin in hepatocellular carcinoma cells^64^. In the hematological context, miR-26 is downregulated in Burkitt and Follicular lymphoma^65^. Furthermore, the ectopic expression of miR-26 in Diffuse Large B-cell Lymphoma (DBCL) cells decreased cell proliferation and increased apoptosis^66^.

The miR-15 family consists of three bicistronic clusters: miR-15a/16-1, miR-15b/16-2 and miR-497/195, the latter not required for hematopoietic cell function and with restricted relevance for embryonic development and healthy lifespan^67,68^. miR-15 and miR-16 were the first miRNAs to be associated with cancer acting mainly as oncosuppressors^69^. Indeed, the miR-15a/16-1 cluster located at chromosome 13q14.3 is frequently deleted or downregulated in B cell malignancies, such as chronic lymphocytic leukemias (CLLs)^69^, multiple myeloma^70^ and in a fraction of Acute Myeloid Leukemia (AML) patients^71,72^. These miRNAs have been described to induce apoptosis, through the inhibition of prosurvival genes as Bcl-2 and directly or indirectly to deregulate genes involved in cell cycle progression^55,73^.

Our specific interest was to evaluate if these miRNAs are responsible for Morgana downregulation in the BM of CML patients. Our analysis on a cohort of 12 MDS/MPN with neutrophilia and 19 Ph + CML patients indicates that only miR-15a negatively correlates with Morgana expression, suggesting its relevance in regulating its levels in this specific context. Instead, miRNAs 15b, as well as miRNAs 26a and b, exhibit no significant anti-correlation.

Intriguingly, the loss of expression of miR-15/16 and the consequent overexpression of their targets have been described as critical features in the transition from chronic phase to blast crisis in CML patients^74,75^. Protein downregulation through miRNA expression is a reversible mechanism that allows adaptation to different phases of the pathology. It is possible to speculate that the overexpression of miR-15, and consequently low Morgana levels, favors the onset of the disease and sustains the chronic phase, by inducing ROCK overactivation and therefore myeloid cell proliferation and genomic instability^3,11,16^. At the opposite, during the progression to blast crisis, malignant cells may benefit more from Morgana overexpression, as well as, other miR-15/16 targets, such as Bmi-1, ROR1, and Bcl-2^74^. Indeed, Morgana is able to act also as a proto-oncogene as it is frequently found overexpressed in different types of tumors, where it sustains AKT and NF-κB activity^9,12^. Unfortunately, we were unable to analyze Morgana expression levels in patients during blast crisis, as these samples are extremely rare due to the success of TKI therapy in Ph + CML and the rarity of MDS/MPN with neutrophilia.

It is conceivable that several mechanisms may regulate Morgana expression levels and that regulation must be cell type-dependent and may vary among patients and stages of the pathology. Thus, miR-15 and miR-26 family members are likely to be only some of the players involved. To clarify the mechanism by which Morgana is downregulated in CML patients remains a crucial point to understand the onset and progression of this pathology and, consequently, to choose the best therapy to avoid resistance and design therapeutic interventions for MDS/MPN with neutrophilia patients, for which overall survival remains very poor^10,31–33^.

## Methods

### Cell culture and antibodies

HEK293T cells were purchased from the American Type Culture Collection (ATCC). Cells were cultured in DMEM, 10% FBS, 5% PS and were incubated at 37 °C, 5% CO_2_. Fetal bovine serum (FBS) and penicillin–streptomycin (PS) were obtained from Thermo Fisher Scientific (Carlsbad, CA, USA). Western blotting was performed using the following primary antibodies: anti-HSP90α/β (sc-13119; 1: 1000), anti-LIMK1 (Cell Signaling, #3842), anti-phospho-LIMK1 (Thr508)/LIMK2 (Thr505) (Cell Signaling, #3841) and anti-Morgana (P1/PP0, 1 μg/ml). P1/PP0 is a mouse monoclonal antibody obtained in our laboratory by immunizing mice with a Glutathione-S-transferase (GST)-mouse Morgana recombinant fusion protein produced in E. coli. Antibody reactivity and specificity were characterized by Western blotting, immunoprecipitation, immunofluorescence and immunohistochemistry. The P1/PP0 antibody recognizes both mouse and human Morgana^3^.

### Fluorescence in situ hybridization (FISH)

We produced the FISH probe for Morgana from BAC RP11-1129K7 which covers the region 11q14.3 of the CHORDC1 gene. Cells containing BAC RP11-1129K7 (Invitrogen) were expanded overnight in LB medium supplemented with chloramphenicol. The BAC was extracted using Midiprep Kit JET Star 2.0, according to manufacturers’ instructions. Briefly, DNA was extracted using a lysis buffer, precipitated and purified by using isopropanol and cold ethanol 75%. BAC DNA was quantified using a NanoDrop ND-1000 Spectrophotometer and 250 ng was labeled with biotin-14-dCTP and random primers from the BioPrime® DNA Labeling System, Invitrogen. The biotinylated probe was purified using illustra MicroSpin G-50 Columns, GE Healthcare. It was then precipitated and preannealed with 1 µl of Salmon Sperm DNA Solution (Life Technologies) and 30 µl of Human Cot-1 DNA® (Life Technologies). The probe was precipitated with 8 µl of sodium acetate 3 M and 160 µl of ethanol at − 20 °C overnight, then it was washed by cold ethanol 75% and dried. The probe was resuspended in the LSI Buffer (Abbott). The centromere-specific probe (CEP11 Spectrum Green, Vysis Abbott) for chromosome 11 was used as technical control. 10 µµl of solution composed by 1 µl of Morgana probe, 1 µl of CEP11 and 8 µl of LSI buffer (Abbott) were used on each chromosome spread slide. Chromosome spreads in metaphase and interphase were obtained by culturing cells with 0.2 µg/ml of Colcemid (Invitrogen) overnight or for 2 h. Cells were incubated with a hypotonic solution of potassium chloride 0.075 M at 37 °C for 20 min and then a cold fixative solution of methanol: acetic acid (3:1) was added. Chromosome preparations were spread on slides and dried by passages in ethanol series. The hybridization program was set to 75 °C for 5 min and 37 °C for 20 h (ThermoBrite, Abbott Molecular). After washing in 2 × SSC 0.3% NP40 solution at 73.5 °C and saturation, the slides were incubated with streptavidin red-conjugated antibody for 30 min. DNA was labelled with DAPI. All metaphases and interphases on the slide were observed using an ApoTome ZEISS microscope and Carl Zeiss™ AxioVision Rel. 4.8.2 Software (https://www.micro-shop.zeiss.com/).

### Western Blot

Cells were washed in cold phosphate-buffered saline and lysed in a buffer containing 20 mM Tris (pH 7.5), 150 mM NaCl, 1 mM ethylenediaminetetraacetic acid, 1 mM ethylene glycol-bis(β-aminoethyl ether)-N,N,N′,N′-tetraacetic acid, 1% Triton X-100, 1 mM β-glycerophosphate, 1 mM orthovanadate and Protease Inhibitor Cocktail (Sigma Aldrich, Saint Louis, MO). Total protein extracts were analyzed by Western blotting and detected using the chemiluminescent reagent LiteAblot (Euroclone, Milano, Italy). Band intensities were quantified using Quantity One software version 4.6.9 (https://www.bio-rad.com/).

### miR precursors transfection

The following miR precursors were used: Pre-miR miRNA Precursor Negative Control (AM17110), Pre-miR miRNA Precursor Hsa-miR-15a (PM10235), Pre-miR miRNA Precursor Hsa-miR-15b (PM10904), Pre-miR miRNA Precursor Hsa-miR-26a (PM10249), Pre-miR miRNA Precursor Hsa-miR-26b (PM12899), all from Applied Biosystems (Foster City, CA). To obtain transient pre-miR expression, cells were plated at 50% confluency and transfected 24 h later using Lipofectamine2000 reagent (Invitrogen Life Technologies) with 75 nM pre-miR. Overexpression of miRNAs-15a and -26a was performed using the pLemiR-tRFP vector (GE Healthcare, Little Chalfon, UK) lentiviral constructs. The genomic regions containing the murine pre-miRNA-15a or -26a were amplified by PCR and cloned under the control of a CMV promoter into pLemiR-tRFP vector. HEK293T packaging cells were seeded at 40% confluence in DMEM 10% FBS and incubated for 24 h at 37 °C, 5% CO_2_. The following day, packaging cells were co-transfected, using Effectene reagent (Qiagen), with the transfer vectors (pLemiR or pLemiR-15a or pLemiR-26a) the packaging vectors (pRSV.REV) and the envelope vector (pMD2VSV-G). On the subsequent day, the medium was changed and approximately 48 h after transfection the lentivirus-containing medium was collected and passed through a 0.22-μm filter. The virus was divided into aliquots and stored at − 80 °C. Virus solutions supplemented with 8 μg/mL sequabrene (Sigma Aldrich, Saint Louis, MO) were used to infect HEK293T cells.

### Real time-PCR

Total RNA from cells or human BM samples was isolated using TRIzol® Reagent (Invitrogen Life Technologies, Carlsbad, CA) according to the manufacturer’s instructions. All RNA quantifications were performed using the NanoDrop-1000 spectrophotometer (Nanodrop, Wilmington, DE). RNA was reverse transcribed using Applied Biosystem kits. qRT-PCRs for detection of miRNAs were performed with the indicated TaqMan® MicroRNA Assays (Applied Biosystems, Foster City, CA) on 10 ng total RNA according to the manufacturer’s instructions. Gene expression analysis of CHORDC1 was performed using the TaqMan Gene Expression Assay (Applied BioSystems). Quantitative normalization was performed on the expression of U44snoRNA for miRNAs and on 18S for CHORDC1. The relative expression level between samples was calculated using the comparative delta CT (threshold cycle number) method (2^−ΔΔ*C*T^) with a control sample as reference point. As defined previously, patient BMs were considered to express low Morgana when CHORDC1 mRNA level was less than half of the mean of the level in normal BMs (n = 10). We demonstrated that using this threshold we obtained 90% correlation with low Morgana level as defined by immunohistochemistry analysis^11^.

### Mutagenesis

The 3’UTR of the human CHORDC1/Morgana gene was obtained by PCR using genomic DNA as template and the primer pair F (5′-AGTGGGAGATGGAAGGAAGG-3′) and R (5′-ACAAAAGAAAGATGAGAGGCACA-3′) with the addition at the 5′ of the SpeI (on F) and HindII (on R) recognition sequence. The PCR product was cloned into pGEM vector (Promega, Madison, WI). The sequence was mutated at the miR-15a/b binding site with a four-base substitution by using the QuikChange Site-Directed Mutagenesis kit (Stratagene, La Jolla, CA). The mutagenized sequence was then sequenced, excised using SpeI and HindIII and subcloned into the pMIR-REPORT™ System (Applied Biosystem, Foster City, CA).

### Luciferase assay

The 3′UTR of CHORDC1, previously generated by PCR amplification of the full length 3′UTR from a plasmid containing the human cDNA sequence of CHORDC1, was inserted into the pMIR REPORT™ luciferase vector (Ambion, Austin, TX). miR-15a and miR-15b binding site in the 3′UTR was mutagenized using the QuickChange Site-Directed Mutagenesis kit (Stratagene, Cedar Creek, TX) according to the manufacturer’s instructions. 6.5 × 10^4^ cells were co-transfected with 50 ng of the pMIR REPORT™ (Ambion, Austin, TX), Firefly Luciferase constructs containing WT or specific miR-15a/b binding site-mutated sequences, 20 ng of pRL-TK Renilla Luciferase as a transfection efficiency control (Promega, Madison, WI) and 75 nM of the indicated pre-miR using Lipofectamine^TM^2000 (Invitrogen Life Technologies, Carlsbad CA). Lysates were collected 48 h after transfection and Firefly and Renilla Luciferase activities were measured with a Dual-Luciferase Reporter System (Promega, Madison, WI).

### Patient samples

Primary leukemia cells were obtained from the BM of patients with MDS/MPN with neutrophilia and Ph + CML in chronic phase after appropriate informed consent and Institutional Review Board approval (Institutional Ethics Committee Approval # 81/2011). All samples were collected at diagnosis before initiation of treatment. The research was performed in accordance with relevant guidelines and regulations. BM samples were processed for RNA extraction and quantitative Real Time-PCR.

### Correlation analysis

For CLL study, two GEO series (belonging to the same GSE51529 GEO superSeries) have been analyzed: GSE51527—MicroRNA Profile and GSE51528—Gene Expression**.** This superSeries of microarray experiments contains the gene and miRNA paired expression profiles of purified B-cell chronic lymphocytic leukemia (B-CLL) cells obtained from 210 patients (Binet stage A); both single series contain a higher number of patients, but some of them cannot be matched. TCGA diffuse large B-cell lymphoma data have been selected to analyze Morgana-miRNA correlation. The common samples between the gene and miRNA datasets are 47 (RNA_Seq data are annotated to Human Mar. 2006 (NCBI36/hg18). Computational analyses were performed by GenoBiToUs Genomics and Bioinformatics Service (University of Turin).

### Statistical analysis

For statistical analyses, significance was tested using a two-tailed Student’s t-test or, when required, one- or two-way ANOVA with Bonferroni’s correction. A minimum value of *p* < 0.05 was considered to be statistically significant. Statistical analyses were performed using GraphPad Prism version 8.0.2 (https://www.graphpad.com/)**.**

### Supplementary Information

Supplementary Figures.

Supplementary Information 1.

Supplementary Table S1.

Supplementary Table S2.

## Data Availability

All the data generated and/or analyzed during the current study are included in this article.
