# Supplementary material for: miR-15a targets the HSP90 co-chaperone Morgana in chronic myeloid leukemia
Source: Sci Rep. 2024 Jul 2;14:15089. doi: 10.1038/s41598-024-65404-7 (PMC11220062; doi:10.1038/s41598-024-65404-7)
Supplement: Supplementary file 1 — Supplementary Figures. [file 41598_2024_65404_MOESM1_ESM.pdf]

Supplementary Figure 1

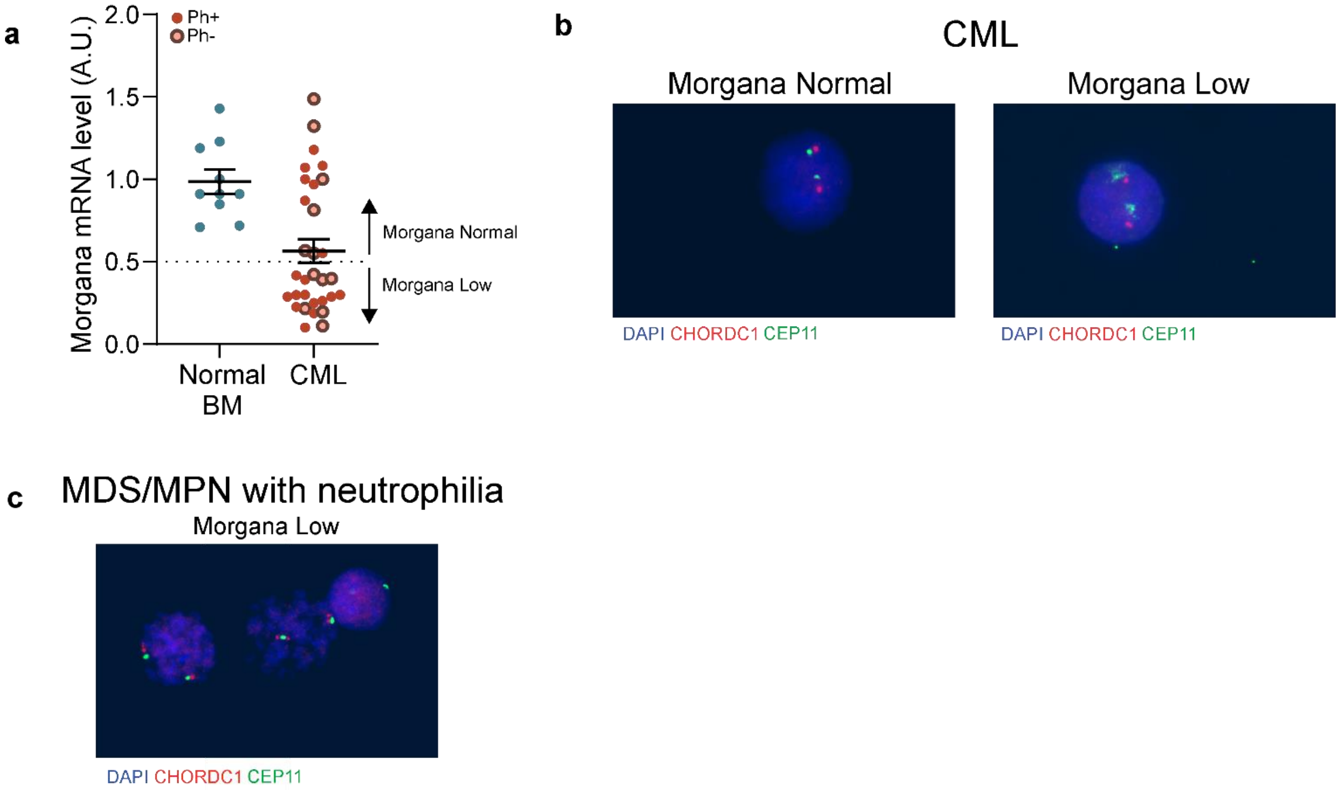

Supplementary Figure S1. Morgana gene (CHORDC1) is not deleted in CML and MDS/MPN with neutrophilia cells. (**a**) Morgana expression levels assessed by qRT-PCR in normal bone marrows (n=10) and in CML samples (Ph+ n=19, Ph- n=12). (**b**) Representative images of FISH from BM cells of Morgana normal (n=4) and Morgana low (n=5) patients affected by Ph+ CML. Two chromosome 11 (marked by CEP11, green points) and two CHORDC1 alleles (red points) are present in each cell. (**c**) Exemplificative images of FISH on interphases from BM cells of Morgana low patients affected by MDS/MPN with neutrophilia (n=3).

Supplementary Figure 2

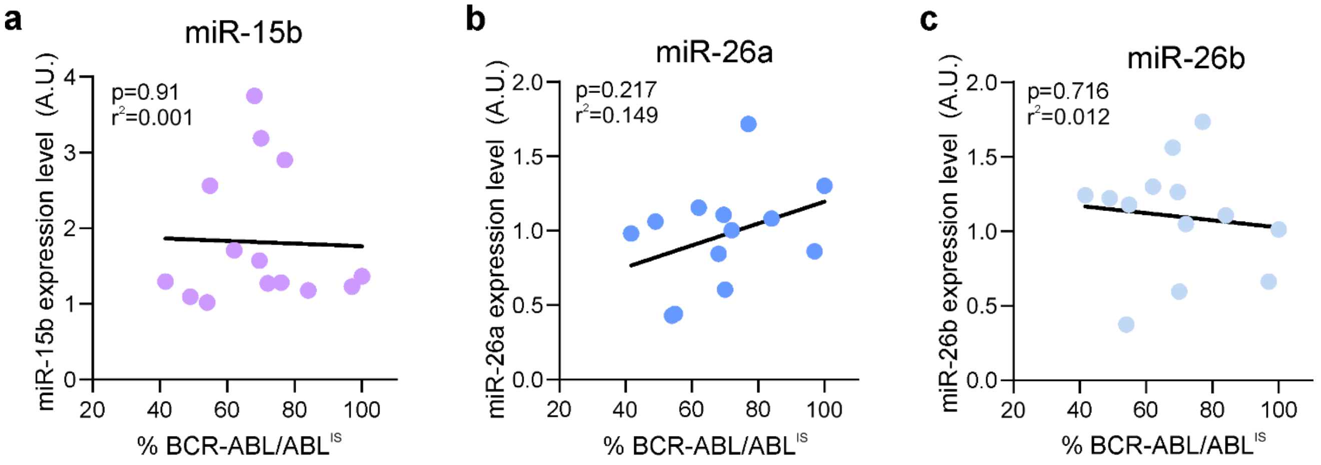

Supplementary Figure S2. Correlation between miRNAs expression and BCR-ABL. Correlation between miR-15b (**a**), miR-26a (**b**), miR26b (**c**) and BCR-ABL expression (International Scale) in Ph+ CML patients.
