# Supplementary material for: miR-15a targets the HSP90 co-chaperone Morgana in chronic myeloid leukemia
Source: Sci Rep. 2024 Jul 2;14:15089. doi: 10.1038/s41598-024-65404-7 (PMC11220062; doi:10.1038/s41598-024-65404-7)
Supplement: Supplementary file 2 — Supplementary Information 1. [file 41598_2024_65404_MOESM2_ESM.pdf]

Supplementary Information

Figure 2c

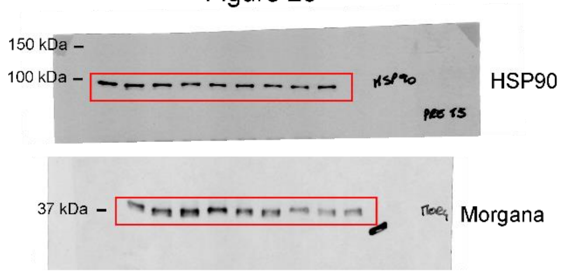

Figure 2d

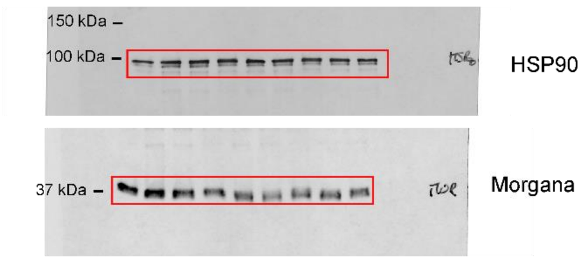

Figure 2g

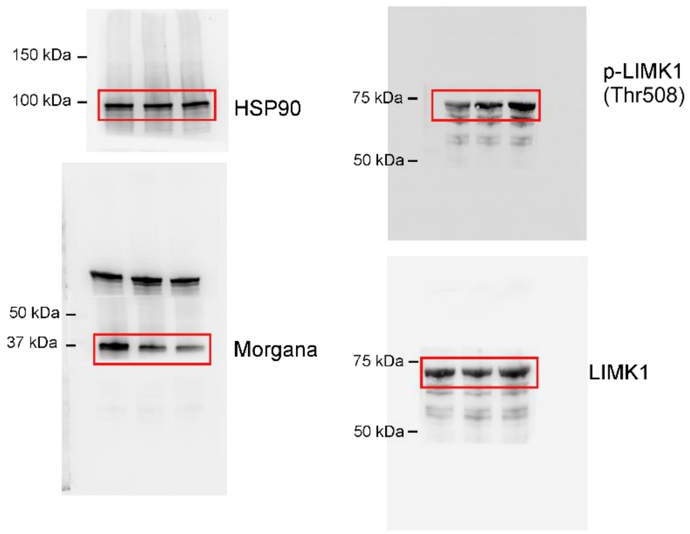

Supplementary Information. Uncropped Images of Western Blot present in Fig. 2 c, d and g. In Fig 2g P-LIMK1 and LIMK1 antibodies are both produced in rabbit. To avoid misinterpretation due to imperfect stripping, we probed two distinct membranes blotted with the same HEK293 total extracts with the two antibodies. We used HSP90 antibody as loading control on both membranes. The membranes were cut before hybridization to probe the samples with multiple antibodies.
